# Genome-Wide Transcriptional and Post-transcriptional Regulation of Innate Immune and Defense Responses of Bovine Mammary Gland to *Staphylococcus aureus*

**DOI:** 10.3389/fcimb.2016.00193

**Published:** 2016-12-26

**Authors:** Lingzhao Fang, Yali Hou, Jing An, Bingjie Li, Minyan Song, Xiao Wang, Peter Sørensen, Yichun Dong, Chao Liu, Yachun Wang, Huabin Zhu, Shengli Zhang, Ying Yu

**Affiliations:** ^1^Key Laboratory of Animal Genetics, Breeding and Reproduction, Ministry of Agriculture & National Engineering Laboratory for Animal Breeding, College of Animal Science and Technology, China Agricultural UniversityBeijing, China; ^2^Department of Molecular Biology and Genetics, Center for Quantitative Genetics and Genomics, Aarhus UniversityTjele, Denmark; ^3^Key Laboratory of Genomic and Precision Medicine, Beijing Institute of Genomics, Chinese Academy of SciencesBeijing, China; ^4^Department of Animal Biotechnology and Reproduction, Institute of Animal Sciences, Chinese Academy of Agricultural SciencesBeijing, China

**Keywords:** *Staphylococcus aureus*, bovine mastitis, innate immune responses, transcriptome, miRNAome

## Abstract

*Staphylococcus aureus* (*S. aureus*) is problematic for lactating mammals and public health. Understanding of mechanisms by which the hosts respond to severe invasion of *S. aureus* remains elusive. In this study, the genome-wide expression of mRNAs and miRNAs in bovine mammary gland cells were interrogated at 24 h after intra-mammary infection (IMI) with high or low concentrations of *S. aureus*. Compared to the negative control quarters, 194 highly-confident responsive genes were identified in the quarters with high concentration (10^9^ cfu/mL) of *S. aureus*, which were predominantly implicated in pathways and biological processes pertaining to innate immune system, such as cytokine-cytokine receptor interaction and inflammatory response. In contrast, only 21 highly-confident genes were significantly differentially expressed in face of low concentration (10^6^ cfu/mL) of *S. aureus*, which slightly perturbed the cell signaling and invoked corresponding responses like vasoconstriction, indicating limited perturbations and immunological evading. Additionally, the significant up-regulations of bta-mir-223 and bta-mir-21-3p were observed in the quarters infected by high concentration of *S. aureus*. Network analysis suggested that the two miRNAs' pivotal roles in defending hosts against bacterial infection probably through inhibiting *CXCL14* and *KIT*. The significant down-regulation of *CXCL14* was also observed in bovine mammary epithelial cells at 24 h post-infection of *S. aureus* (10^8^ cfu/mL) *in vitro*. Integrated analysis with QTL database further suggested 28 genes (e.g., *CXCL14, KIT*, and *SLC4A11*) as candidates of bovine mastitis. This study first systematically revealed transcriptional and post-transcriptional responses of bovine mammary gland cells to invading *S. aureus* in a dosage-dependent pattern, and highlighted a complicated responsive mechanism in a network of miRNA-gene-pathway interplay.

## Introduction

Mastitis, generally defined as inflammatory changes in the mammary gland with an invading microbial agent, has been considered as one of the most prevalent and economically significant diseases in dairy sector worldwide over the last decades (De Oliveira et al., 2000; Bradley, 2002; Halasa et al., 2007; Middleton et al., 2014), which is not only a concern for animal welfare but also a risk for public health as milk is an essential source of human nutrition. Among various mastitis-causing pathogens, gram-positive *Staphylococcus aureus* (*S. aureus*) is one of the most common etiological bacteria (Bradley, 2002) and is also the most prevalent pathogen relevant to human mastitis (Amir et al., 1999). Epidemiological studies in humans reported that up to a third of all lactating women suffered from mastitis (Foxman et al., 2002).

*S. aureus* often causes subclinical and chronic mastitis affecting all lactating mammals due to its resistance to antibiotic treatments and its capability to evade the host's innate and adaptive immune responses (Fox and Gay, 1993; Bradley, 2002; Contreras and Rodríguez, 2011; Spaan et al., 2013). In addition, *S. aureus*-induced bovine mastitis is contagious and can be readily transmitted among udder quarters and animals during the milking process (Fox and Gay, 1993). Therefore, mastitis induced by *S. aureus* is particularly difficult to be prevented and eliminated, leading to a huge economic loss and overuse of antibiotics. Furthermore, it has been documented that oxacillin-susceptible *mecA*-positive *S. aureus* (OS-MRSA) is usually found in the milk of cows suffering from mastitis, which may be very prone to enhancing methicillin-resistant *S. aureus* (MRSA) under antibiotic selection due to the possession of *mecA* gene (Holmes and Zadoks, 2011; Pu et al., 2014). Keeping in view of the strong need to decrease the use of antibiotics and further enhance food safety as well as animal welfare, effective genetic improvement of immune response through precise genomic selection for mastitis resistance in dairy animals has been considered as a prophylactic and economical approach (Keirn et al., 2001; Tao and Mallard, 2007; Pighetti and Elliott, 2011; Sordillo, 2011).

It has been well-established that a better understanding of the genetic and biological basis of complex diseases could benefit their genomic prediction and development of appropriate control strategies (Hayes et al., 2010; Snelling et al., 2013; Edwards et al., 2016; Sarup et al., 2016). Therefore, the innate defense mechanism of mammary gland against invading pathogens during the early time of infection is urgently needed to be clarified for controlling mastitis (Oviedo-Boyso et al., 2007; Sordillo, 2011; Thompson-Crispi et al., 2014), which could include identifying and characterizing the involved network of genes, pathways and post-transcriptional regulatory elements (e.g., miRNA). Although the transcriptional (i.e., mRNA) response of the bovine mammary gland to intra-mammary infection (IMI) with *S. aureus* has been studied previously (Tao and Mallard, 2007; Lutzow et al., 2008; Jensen et al., 2013), revealing many innate immune relevant genes (e.g., *IL1B, IL6*, and *IFNG*) and pathways (e.g., NF-kappa B signaling pathway and Cytokine-cytokine receptor interaction), few studies have explored how miRNA modulates the innate immune response of bovine mammary gland to the presence of *S. aureus in vivo*. It has been well-proposed that miRNA, an endogenous non-coding small RNA, plays a pivotal role in the regulation of the innate immunity through post-transcriptionally regulating relevant gene expression in many species (Lindsay, 2008; Bi et al., 2009). This gap of knowledge is needed to be filled to understand the transcriptional and post-transcriptional responses of bovine mammary gland cells to *S. aureus* infection, and to detect causative genes and pathways for developing therapeutic agents and improving genomic selection for bovine mastitis.

The two goals of this study are: (1) to detect the innate immune responses and the global networks of genes, pathways and miRNAs that were activated in bovine mammary gland at 24 h post IMI with *S. aureus*; (2) to identify powerful candidate genes associated with bovine *S. aureus* mastitis for follow-up functional studies.

## Materials and methods

### Samples

All the following procedures involving animals were approved by the Animal Welfare Committee of China Agricultural University, Beijing, China, and animal experiments were conducted in strict accordance with regulations and guidelines established by this committee.

As each udder quarter within a cow is generally considered as an independent anatomical structure, the utilization of within-animal control is well-accepted as a common practice (Lutzow et al., 2008; Mitterhuemer et al., 2010; Buitenhuis et al., 2011; Jensen et al., 2013), and it also agrees with the ethical framework of 3Rs (Reduction, Replacement, and Refinement) for carrying out animal experiments. In this study, six samples from six quarters of two Chinese Holstein cows in their early first lactation were involved. The fresh milk from each udder quarter of the studied cows was detected for major or minor mastitis-causing pathogens to ensure that the cows were free from infection by using the previously reported methods (Wang et al., 2014). The cows were then evaluated for their general health status based on rectal body temperature and milk somatic cell count (SCC). The milk SCC for each studied udder quarter was determined with a Fossomatic 5000^*^(FOSS Electric, Hillerod, Denmark) (range 1–9999 × 10^3^ cells/mL) at 3 weeks, 3 and 0 days before disease challenge, respectively.

### *S. aureus* isolation and inoculum with gradient dosage

The *S. aureus* used in this study was isolated from the milk of Chinese Holstein cows with *S. aureus*-induced mastitis, which has been described previously (Wang et al., 2014). Briefly, a total of 3 mL collected milk was mixed into 30 mL of trypticase soy broth (TSB, Beijing Land Bridge Technology Ltd., Beijing, China) containing 75% NaCl. After 18–24 h incubation at 37°C, a total of 10 mL culture was placed onto Baird-Parker agar plates (BPA, Beijing Land Bridge Technology Ltd.) with tellurite and 5% egg yolk. Following incubation for 24 h at 37°C, two coagulase-positive colonies of each sample (black colonies surrounded by 2–5 mm clear zones) were transferred to trypticase soy agar plates (TSA, Beijing Land Bridge Technology Ltd.) for further purification. Finally, colonies were confirmed as *S. aureus* using specific PCR detection on the thermonuclease gene (*nuc, S. aureus* specific).

The concentration of *S. aureus* was determined by dilution plate counting method (Atlas, 2010). Prior to infection, a total of 50 μL *S. aureus* was transferred to a tube of trypticase soy broth (5 mL, Beijing Land Bridge Technology Ltd., Beijing, China) and incubated for 24 h at 37°C in a 200 rpm-shaking incubator. Next, *S. aureus* was diluted six gradients successively with 0.9% sterile, pyrogen-free saline. Then 100 μL diluent was transferred into plate count agar (PCA, Beijing Land Bridged Technology Ltd., Beijing, China) and spread with a glass spreader. Subsequently, the agar plates were incubated at 37°C for 18–24 h. After PCA plate culture of *S. aureus* diluent, the number of *S. aureus* colonies was counted. Each diluent PCA was conducted in triplicate. Finally, bacteria were diluted in the DMEM medium to obtain 1 × 10^6^ cfu/mL and 1 × 10^9^ cfu/mL, respectively. The diluted *S. aureus* were stored at 4°C shortly for infection.

### *S. aureus* challenge, milk SCC, and body temperature testing

Except for the front left udder quarters of the two studied cows, the remaining three quarters for each cow were involved in the treatments. The rear left and right udder quarters for each cow were inoculated with 10 mL low- (10^6^ cfu/mL) and high- (10^9^ cfu/mL) concentration of *S. aureus* through teat canal after morning milking respectively, while the front right quarter received 10 mL 0.9% sterile pyrogen-free saline at the start of the trial as placebo. The rectal body temperature was recorded at 0, 6, 12, 18, and 24 h post *S. aureus* challenge respectively. Around 10~30 mL milk sample was aseptically collected from each udder quarter at the same time points (i.e., 0, 6, 12, 18, and 24 h) throughout the trial. The milk SCC was then determined using Fossomatic 5000^*^(FOSS Electric, Hillerod, Denmark). The infected cows were kept isolated from the herd during the entire experiment.

### Udder biopsies collection

Udder biopsies were sampled from the infected and control quarters of the studied cows at 24 h after IMI with *S. aureus* according to a previously published procedure (Buitenhuis et al., 2011). Briefly, the skin surface of each udder quarter was thoroughly washed and dried. After a mild sedation injection and local anesthetic, a 0.3–0.8 cm long incision was made in the skin area of the middle of each quarter with a scalpel. The udder biopsies were sampled at the incision point with a biopsy pistol that was developed for collecting biopsies in human. After sampling, the sterile cloth tampons were then pressed on the incision areas to stop bleeding and a prophylactic antibiotic treatment was administered for each cow. The collected udder biopsies were immediately stored in RNAstore reagent DP408 (Tiangen Biotech, Co., LTD., Beijing, China) and sent to the laboratory for RNA extraction.

### RNA extraction and quality assessment

The TRIzol reagent (Invitrogen, Carlsbad, CA) was used to isolate the total RNA from each udder biopsies sample. The contamination of DNA in the total RNA was removed using RNase-free DNase I (New England Biolabs). The degradation of RNA was checked on 1% agarose gels. The purity of RNA was monitored on a NanoPhotometer® spectrophotometer (Implen, CA, USA). The concentration and integrity of RNA were assessed using A Qubit® RNA Assay Kit in Qubit®2.0 Fluorometer (Life Technologies, CA, USA) and the RNA Nano 6000 Assay Kit of the Bioanalyzer 2100 system (Agilent Technologies, CA, USA), respectively. All the six RNA samples had a RNA integrity number larger than 9.

### Transcriptome library construction and sequencing

An equal amount (3 μg) of total RNA per sample was used to construct RNA-Seq libraries. Sequencing libraries were generated according to the manufacturer's protocol using the IlluminaTruSeq™ RNA Sample Preparation Kit (Illumina, San Diego, CA, USA). Briefly, mRNA was purified and enriched from total RNA using ploy-T oligo-attached magnetic beads before fragmentation *via* divalent cations (zinc) and heat hydrolysis. First-strand cDNA was synthesized using random oligonucleotides and SuperScript II; Second-strand cDNA was subsequently generated using DNA polymerase I and RNase H before end repairing. After adenylation of 3′ ends of double-stranded cDNA, Illumina PE adapter was ligated. To select cDNA fragments of approximately 200 bp in length, AMPure XP system (Beckman Coulter, Beverly, USA) was applied to purify the library fragments. Size-selected fragments were PCR-amplified using KAPA HiFi polymerase (Kapa Biosystems, Boston, MA) before finally sequencing on an Illumina HiSeq2000 platform (Illumina, San Diego, CA, USA).

For miRNA sequencing, an equal amount (3 μg) of total RNA for each of the six samples was used to build miRNA libraries using the TruSeq Small RNA Sample Preparation kit (Illumina, San Diego, CA, USA) following the manufacture's protocol. PCR amplification was performed for 12 cycles. Agilent 2100 Bioanalyzer was then used for quality control. Totally, six small RNA libraries were constructed after gel purification for selecting fragments of 140–160 bp in length. Sequencing was conducted on the Genome Analyzer IIx system (Illumina) using the TruSeq SR Cluster Kit v2 (Illumina) and TruSeqTM SBS kit v5-GA (36 cycles, Illumina).

All the raw sequencing data in this study have been submitted to the NCBI's Sequence Read Archive (SRA) with the accession number SRP073432.

### Quality control for raw data

For both RNA-seq and miRNA-seq, reads with more than 10 Ns and low-quality reads (i.e., more than fifty percent of the reads with a quality score of less than 10 or read length < 30) were removed from the raw data to obtain clean data (clean reads). The quality of clean data was then evaluated using FASTQC v0.10.1 (http://www.bioinformatics.babraham.ac.uk/projects/fastqc/). All the downstream analyses were conducted on the clean data. A summary of the bioinformatics analyses strategy used in this study was outlined in Figure S1.

### Reads alignment and differential expression analysis for RNA-Seq

Two biological replicates for each experimental condition were used in this study. The impact of varying number of biological replicates on the detection of differentially expressed genes was investigated by a previous study (Rapaport et al., 2013), which demonstrated that with most bioinformatics approaches, over 90% of differentially expressed genes (DEG) at the top expression levels could be captured by using two biological replicates. In this study, two of the most frequently used approaches were applied to identify highly-confident DEGs, namely Cufflinks-Cuffdiff2 (Trapnell et al., 2012) and edgeR (Robinson et al., 2010).

For Cufflinks-Cuffdiff2 approach, Bowtie2 v2.1.0 (http://bowtie-bio.sourceforge.net/bowtie2/manual.shtml) was applied to create an index of the bovine reference genome (UMD3.1) obtained from Ensembl (ftp://ftp.ensembl.org/pub/release-73/fasta/bos_taurus/dna/). TopHat v2.0.9 (http://ccb.jhu.edu/software/tophat/index.shtml) was then employed to align paired-end clean data of each sample to the reference genome with default settings. Only reads mapped to a unique location were used for following analyses. Cufflinks v2.1.1 (http://cufflinks.cbcb.umd.edu/) was applied to assemble TopHat's read alignments, in order to improve the accuracy of inferring differentially expressed transcripts. Cuffdiff2 was then used to detect DEGs between different conditions. The statistical model implemented in Cuffdiff2 assumes that the reads count distribution follows beta negative binomial distribution. The FPKM (fragments per kilobase per million mapped fragments) normalization procedure was used to account for different gene lengths and different sequencing depths across RNA-Seq libraries. The Benjamini-Hochberg method (FDR) was then employed to correct for multiple testing.

For edgeR method, the index of reference genome was also firstly created by build-index function implemented in Rsubread package with default options (Liao et al., 2013). Sequence reads obtained for each sample were then aligned separately to the bovine reference genome assembly using a sensitive and efficient mapping program based on seed-and-vote algorithm implemented in the Rsubread package in R/Bioconductor. The number of reads mapped to each of the 24,616 bovine Ensembl genes was counted using the function of Feature-Counts in the Rsubread package under the default setting. Differential expression analysis of genes was conducted using edgeR. The weighted trimmed mean of M-values was used to normalize the count data. A negative binomial generalized linear model (GLM) was applied, as count data follow a non-normal distribution and commonly exhibit a quadratic mean-variance relationship. To ensure stable inference for each gene, an empirical Bayes method was used to squeeze the gene-wise dispersions toward a common dispersion for all genes. Experiment design was modeled by GLM and differential expression was determined using a likelihood ratio test. The statistical test in each analysis was adjusted for multiple testing using the FDR method. Ultimately, genes with FDR < 0.05 and absolute log_2_(fold-change) > 1 from both methods were considered as DEGs.

### Reads alignment and differential expression analysis for miRNA-Seq

The bovine reference genome was firstly indexed using bowtie-build with default options (Trapnell et al., 2012). The deep-sequencing reads were then aligned to the indexed reference genome using bowtie in mapper module in miRDeep2 package (Friedländer et al., 2012), where the reads less than 18 bp or assigned as PCR duplicates or aligned to more than five genomic positions were exquisitely discarded. Perfect alignment without any mismatch was strictly required. The identification of miRNA was also performed using miRDeep2 package, which uses a probabilistic model of miRNA biogenesis to score compatibility of the position and frequency of sequenced RNA with the secondary structure of the miRNA precursor, where the known miRNA precursor and mature miRNA in cattle and human were interrogated, improving the performance of identification in practice. The unknown miRNAs with a filtering criteria of miRDeep2 score ≥ 1, estimated probability of a true positive miRNA candidate > 70%, and a significant Randfold prediction *P*-value were identified as novel miRNAs. The differentially expressed miRNAs between different conditions were excavated by implementing DESeq package (Anders and Huber, 2010). The statistical test for each miRNA was then adjusted for multiple testing using the FDR method, and the miRNAs with FDR < 0.01 and absolute log_2_(fold-change) > 1 were then considered as differentially expressed miRNAs (DE miRNAs).

### Target prediction of miRNA and screening

Given the high false-positive rates for miRNA target prediction in animals (Akhtar et al., 2016), the target genes of DE miRNAs were simultaneously predicted using three most frequently updated algorithms with default settings and no specific prediction threshold (Jens and Rajewsky, 2015), including TargetScan 7.0 (Agarwal et al., 2015), miRDB (Wong and Wang, 2014) and miRmap (Vejnar and Zdobnov, 2012). For TargetScan7.0 and miRmap, the cow was selected as the reference organism, whilst for miRDB, human had to be chosen because of the avoidance of cow database, which was acceptable since both organisms evolutionarily belong to mammals. As it has been well-established that miRNAs mainly down-regulate gene expression through suppressing translation or stimulating mRNA degradation (Cai et al., 2009), the predicted target genes that were identified by at least two approaches were then refined by using the inverse correlation between expression of a miRNA and its target mRNAs (Nam et al., 2009).

### Downstream bioinformatics analysis

The functional enrichment analysis of DEGs was conducted using a hypergeometric gene set enrichment test based on GO and KEGG pathway database, as implemented in web-based tool called KOBAS2.0 (Xie et al., 2011). The clusters with FDR less than 0.05 were determined as statistically significant. GeneMANIA (Warde-Farley et al., 2010) and STRING.10 (Szklarczyk et al., 2014) were used to identify the gene-gene interaction (GGI) network and protein-protein interaction (PPI) network, respectively. The GGI network was constructed based on six types of evidence (co-expression, co-localization, pathway, shared protein domains, physical interaction, and association network) from GeneMANIA Human database, while the PPI network between the proteins was examined based on four types of evidence from STRING.10 database: Experimental, text-mining, co-expression, and databases. The detailed information concerning GeneMANIA and STRING.10 were published previously (Warde-Farley et al., 2010; Szklarczyk et al., 2014).

### Real-time quantitative PCR (RT-qPCR) analysis

The cDNA synthesis for each of the six mammary gland tissue samples was performed using PrimeScriptTM RT reagent kit according to the manufacturer's instructions (Takara, Dalian, China). The RT-qPCR reactions were conducted in a final volume of 20 μL with the Roche SYBR Green PCR Kit (Roche, Hercules, CA, USA) according to the manufacturer's protocol. The bovine reference gene *GAPDH* was used as the internal standards to adjust the input of cDNA and to normalize the expression of target genes. Triplicate RT-qPCRs were carried out on each cDNA and the average Ct value was used for further analyses. The value of 2^−ΔΔCt^ was calculated as the relative quantification value, and significant differences between conditions were examined by a Student's *t*-test. In order to compare with sequencing data results, log_2_-transformed fold-change was also calculated for RT-qPCR in each comparison.

### Bovine mammary epithelial cell (Mac-T cell) culture and *S. aureus* challenge for *in vitro* validation

Mac-T cells line was a generous gift from Zhejiang University, Zhejiang, Hangzhou, China. Mac-T cells were re-suspended in warm growth medium, and were then cultured up to a maximum of three passages to reduce the risk of aberrant expression caused by extended culturing. Mac-T cells were seeded at 3 × 10^5^ cells/well in six well-plates for *S. aureus* stimulation. At 85% confluence, Mac-T cells were stimulated with *S. aureus* (10^8^ cfu/mL) isolated from subclinical mastitis cows for 3 and 24 h to enable a ratio of 10 bacteria to 1 Mac-T cell (MOI = 10:1). Control group cells were left untreated. Each experimental treatment was conducted in triplicate. Cell lysates were collected and stored at −80°C for RNA extraction. RNA extraction and RT-qPCR for Mac-T cells *in vitro* validation study were performed using the same approaches as above. The primers information for the validation of *CXCL14, IL-8, IL-6*, and *JAK2* genes were shown in Supplementary Table S1.

## Results

### Significant physiological changes induced by IMI with *S. aureus*

Prior to *S. aureus* treatments, cows involved in the present study were examined at normal body temperatures (Table 1). Moreover, all the udder quarters of the studied cows were guaranteed free from major or minor mastitis-causing pathogens, and the milk SCC for each studied quarter was less than 10^5^ cells/mL (Table 2), a widely adopted criterion for normal quarters without inflammation (Smith, 2002). After inoculation of *S. aureus* into the mammary glands, the challenged cows developed severe fever (> 40.0°C) with the highest body temperature at around 41.8°C within 24 h post IMI (Table 1), and showed sharper increases in milk SCC in the udder quarters invaded by *S. aureus* compared to control quarters (Table 2). Of note, compared to udder quarters infected with low concentration of *S. aureus*, the udder quarters infected with high concentration exhibited much stronger physiological responses including udder swelling and milk coagulation that severely obstructed the detection of SCC (Table 2).

**Table 1:** **Overview of the changes in body temperature (°C) for the two studied cows before and after intra-mammary infection with ***S. aureus*****.

	**−21 days**	**−3 days**	**0h**	**6h**	**12h**	**18h**	**24h**
Cow#1	38.4	38.4	38.5	40.8	41.2	41.4	41.8
Cow#2	38.3	38.5	38.2	41.0	41.3	41.5	41.5

**Table 2:** **Overview of the changes in milk somatic cell count (10^**3**^ cells/ml) for each studied udder quarters before and after intra-mammary infection with ***S. aureus*****.

		**−21 days**	**−3 days**	**0h**	**6h**	**12h**	**18h**	**24h**
Control	Cow#1	30	20	19	1030	1968	2020	2441
	Cow#2	34	18	28	1050	1802	1843	2046
Low	Cow#1	16	12	16	5425	8544	9692	8452
	Cow#2	26	12	29	7196	6992	8743	8232
High	Cow#1	20	26	17	>9999	>9999	>9999	>9999
	Cow#2	43	21	31	>9999	>9999	>9999	>9999

### Sequencing and profiling of transcriptomes and miRNAomes in response to infection of *S. aureus*

For RNA-Seq, three disparate sets of transcriptomic profiles were achieved: (I) mock infected quarters (Control); (II) quarters infected with low concentration of *S. aureus* (Low); (III) quarters infected with high concentration of *S. aureus* (High). In total, 51,316,826~66,165,626 paired-end reads of 100 bp in length per library were acquired as raw data. After filtering, an average of 53,781,850 paired-end reads (ranging from 50,013,352 to 64,092,924) still remained for each library, and the averaged uniquely mapping rate was more than 76% across samples (Table S2). Out of the 24,616 bovine annotated genes, an average of 16,746 genes (ranging from 15,825 to 17,272) was examined as expressed across samples.

For miRNA-Seq, an average mapping rate of 61.12% was obtained across samples. The detailed mapping statistics for each sample were summarized in Table S3. Ultimately, 40 novel miRNAs were identified (Table S4), and 385 known mature bovine miRNAs were herein repeatedly recalled and further quantitatively profiled (Table S5).

### Transcriptional alterations of the mammary gland responsive to invasion of *S. aureus*

**Figure 1:**
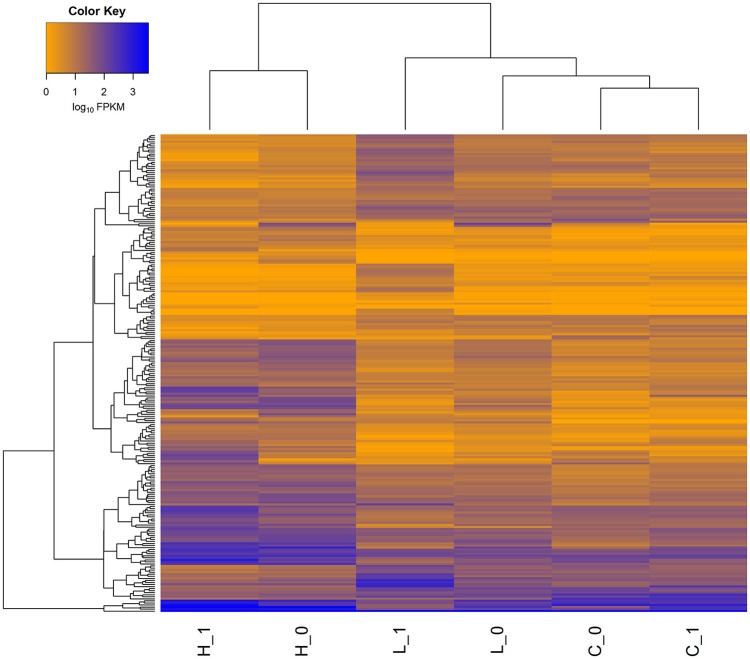
**Heat-map of gene expression for all the differentially expressed genes (DEGs) in three comparisons**. A total of 315 genes was differentially expressed (FDR < 0.05) in three comparisons (i.e., High vs. Control, Low vs. Control, and High vs. Low). H_0 and H_1 represent the two biological replicates in High group, L_0 and L_1 represent the two biological replicates in Low group, while C_0 and C_1 represent the two biological replicates in Control group. The FPKM values of genes (rows) are ordered using hierarchical clustering, and the samples are grouped according to the similarity of expression profiles of genes.

In order to identify highly-confident DEGs, two commonly used approaches were applied. The heat-map of 315 DEGs detected in each of the three comparisons (i.e., High vs. Control, Low vs. Control, and High vs. Low) was illustrated in Figure 1. Noticeably, many more genes were differentially expressed in face to high concentration of *S. aureus*, compared to low concentration. The detailed information of DEGs detected in each comparison was described below.

#### High vs. control comparison

**Figure 2:**
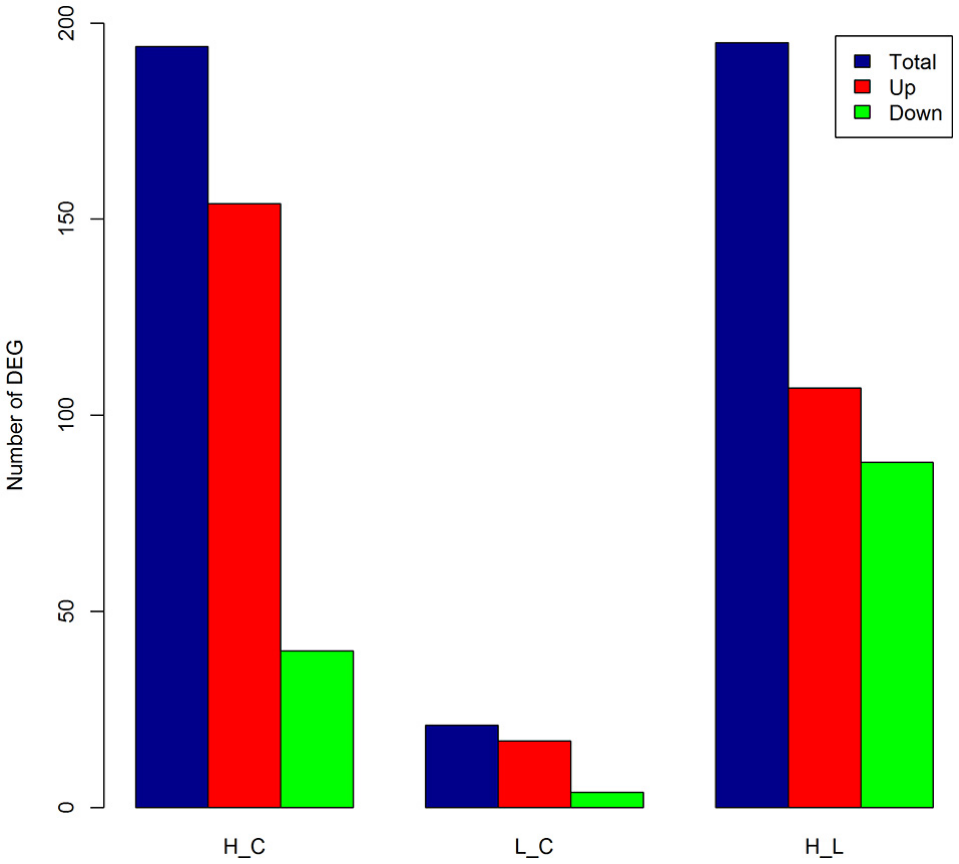
**Number of differentially expressed genes (DEGs) in each comparison**. The number of DEGs [FDR < 0.05 and absolute log_2_(fold-change) > 1] as total (blue), up-regulated (Up, red), and down-regulated (Down, green) in bovine mammary gland at 24 h post IMI with low and high concentrations of *S. aureus*. H_C, L_C, and H_L represent High vs. Control, Low vs. Control and High vs. Low comparisons, respectively.

A total of 194 DEGs (commonly identified by both approaches) was detected (Table S6), among which 154 were up-regulated (Figure 2) with an average log_2_(fold-change) of 3.06 (ranging from 1.43 to 7.98), while 40 DEGs were down-regulated (Figure 2) with an average log_2_(fold-change) of −2.32 (ranging from −3.51 to −1.43). Many classical innate immune-related genes were up-regulated, including genes encoding pro-inflammatory cytokines (e.g., *IL1B, IL-8, IL-17*, and *IL-19*), chemokines (e.g., *CCL3, CCL20*, and *CXCL14*), and their receptors (e.g., *CCRL2, IL1R2, IL1RAP, IL1RL1, IL1RN, IL2RA*, and *IL7R*) as well as tumor necrosis factors (e.g., *TNFAIP3* and *TNFRSF21*). Additionally, genes with antimicrobial functions (e.g., *JAK2, S100A8, S100A12*, and *SLC11A1*) and genes involved in positive regulation of defense responses (e.g., *CD14, DMBT1*, and *TNIP1*) were also up-regulated, showing a clear sign of building up an innate immune defense in the mammary gland against the invasion of high concentration of *S. aureus*. In contrast, multiple genes that were engaged into hormone metabolic processes (e.g., *FOXA1, INHBB, STC2*, and *TTR*), monocarboxylic acid biosynthetic processes and lipid metabolism (e.g., *ALDH1L2, FASN, FGFR4*, and *LPL*) were down-regulated, suggesting that the innate immune responses inhibited metabolism in mammary gland during IMI with high concentration of *S. aureus*.

#### Low vs. control comparison

A mere total of 21 genes was identified as DEGs (Table S7), among which 17 genes were up-regulated (Figure 2), including genes for mesenchymal-epithelial cell signaling (e.g., *TNC* and *WNT2B*), adrenergic receptor and protein kinase C-mediated events (e.g., *CNN1, NR4A3, OXT, RPS15A*, and *SAG*), as well as cell adhesion and death (e.g., *CIDEC, GJA5*, and *MPZ*). Among the four down-regulated genes, only one gene was functionally annotated in the bovine genome, namely Heat Shock Protein Family A (Hsp70) Member 6 (*HSPA6*) with FDR = 2.22 × 10^−3^ and log_2_(fold-change) = −2.48. There were five DEGs shared between High vs. Control and Low vs. Control comparisons, including two annotated genes, *HSPA6* and *RPS15A*. The expression of *RPS15A* in mammary gland was up-regulated in face of both low concentration [log_2_(fold-change) = 2.89] and high concentration of *S. aureus* [log_2_(fold-change) = 3.12]. Of special interest was *HSPA6*, which is related to cellular oxidative stress and heat shock. The expression level of *HSPA6* was down-regulated in the case of invasion of low concentration of *S. aureus*, while reversely up-regulated [log_2_(fold-change) = 2.14] in face of high concentration.

#### High vs. low comparison

A total of 195 DEGs was detected (Table S8), out of which 107 were up-regulated and 88 were down-regulated (Figure 2). The up-regulated genes were mainly involved in classical innate immune, inflammatory responses, and fever induction (e.g., *CD14, CD55, CTSL1, HCK, IL1B, IL6, IL-8, S100A8, S100A12, SOCS3*, and *TNIP1*). Whilst, the down-regulated genes were mainly engaged into regulation of hormone levels (e.g., *CYP1B1, FOXA1*, and *INHBB*), system development (e.g., *CX3CR1, CYP1B1, ELN, INHBA*, and *FOXA1*), angiogenesis (e.g., *AQP1, CD34, COL8A1, SEMA5A*, and *TMEM100*), cell adhesion (e.g., *EGFLAM, ITGB5, FAT4*, and *THBS3*), and cell-cell signaling (e.g., *NEURL, NOV*, and *WNT2B*). Compared to low concentration of *S. aureus*, high concentration of *S. aureus* provoked a more extensive transcriptional alteration, inducing an acute innate immune defense and thereby disturbing normal physiological functions in mammary gland (i.e., udder swelling, fever, and milk coagulation).

### Functional interpretation of mammary transcriptional alterations in response to *S. aureus*

#### High vs. control comparison

**Figure 3:**
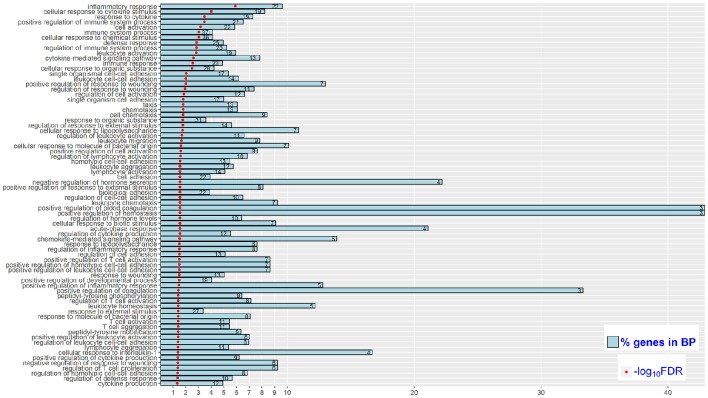
**Significantly enriched (FDR < 0.05) biological processes (BP) in High vs. Control comparison**. Reported are the significance of enrichment [as –log_10_(FDR)]. The % of DEGs relative to all genes in the BP (as % genes in BP), and the number of DEGs in the BP (as the value in each bar).

Functional enrichment analysis of the 194 DEGs based on gene ontology (GO) database revealed 74 significantly enriched Biological Processes (BPs) (FDR < 0.05), which were mainly engaged in inflammatory response, positive regulation of immune system process and cell adhesion (Figure 3). For the Kyoto Encyclopedia of Genes and Genomes (KEGG) pathway overrepresentation analysis, eight significantly enriched pathways were detected (FDR < 0.05) (Table 3), all of which are interconnected and directly involved in innate inflammatory host defense, suggesting that multiple innate immunity and inflammation-related pathways in the mammary gland could be completely mobilized to respond to high concentration of *S. aureus* at 24 h post-infection.

**Table 3:** **Eight significantly overrepresented (FDR < 0.05) KEGG pathways in High vs. Control comparison**.

**KEGG term**	**KEGG ID**	**FDR**	**DEG**
Cytokine-cytokine receptor interaction	bta04060	3.42E-4	*IL17*; *IL6*; *IL2RA*; *KIT*; *IL1RAP*; *IL8*; *CSF3*; *CCL20*; *CXCR2*; *CXCL14*; *IL1R2*; *IL7R*; *IL1B*; *CD40*; *CCR1*; *TNFRSF21*; *LIF*
Legionellosis	bta05134	6.83E-4	*CXCL2*; *IL6*; *IL1B*; *IL8*; *NAIP*; *HSPA6*; *CD14*; *NFKB2*; *NFKBIA*
NF-kappa B signaling pathway	bta04064	1.89E-3	*IL1B*; *TRAF1*; *BCL2A1*; *IL8*; *CD14*; *PTGS2*; *NFKB2*; *TNFAIP3*; *CD40*; *NFKBIA*
Hematopoietic cell lineage	bta04640	3.57E-3	*CD55*; *IL2RA*; *KIT*; *IL6*; *CSF3*; *IL7R*; *IL1B*; *CD14*; *IL1R2*
TNF signaling pathway	bta04668	6.07E-3	*CXCL2*; *IL6*; *TRAF1*; *IL1B*; *SOCS3*; *CCL20*; *PTGS2*; *TNFAIP3*; *NFKBIA*; *LIF*
NOD-like receptor signaling pathway	bta04621	2.85E-2	*IL1B*; *IL6*; *IL8*; *NAIP*; *TNFAIP3*; *NFKBIA*
Chemokine signaling pathway	bta04062	2.85E-2	*HCK*; *JAK2*; *GRO3*; *IL8*; *FGR*; *CCL20 CXCR2*; *CXCL14*; *NCF1*; *CCR1*; *NFKBIA*
Malaria	bta05144	2.85E-2	*SELP*; *IL1B*; *IL6*; *IL8*; *CSF3*; *CD40*

*FDR, false discovery rate; DEG, differentially expressed gene*.

#### Low vs. control comparison

No significantly enriched BP terms or KEGG pathways were observed.

#### High vs. low comparison

The functional enrichment analysis of the 195 DEGs disclosed 29 significantly enriched BP terms, all of which were related to positive regulation of response to wounding and innate immune responses (Table S9). Out of these 29 enriched BP terms, 20 were shared in the High vs. Control comparison, such as inflammatory response (GO: 0006954), cytokine-mediated signaling pathway (GO: 0019221), and acute phase response (GO: 0006953). Additionally, three significantly enriched KEGG pathways were detected, namely malaria (bta05144), hematopoietic cell lineage (bta04640), and legionellosis (bta05144), and all of them were shared in the High vs. Control group. Compared to low concentration of *S. aureus*, more innate immune relevant biological processes and pathways in mammary gland were activated by high concentration of *S. aureus* at 24 h after IMI.

### Post-transcriptional responses in the mammary gland post IMI with *S. aureus*

The expression correlations for all the profiled 385 known mature miRNAs between each pair of samples were calculated as above 0.93 (Figure S2), exhibiting systematically high resemblance across samples. Two DE miRNAs were identified in High vs. Control comparison, namely bta-mir-223 [FDR = 0.001 and log_2_(fold-change) = 3.25] and bta-mir-21-3p [FDR = 0.003 and log_2_(fold-change) = 2.12]. Totally, 16 and 21 target genes were obtained for these two DE miRNAs respectively (Table S10), demonstrating that they might play a central role in defending host from IMI with high concentration of *S. aureus* through exerting immuno-regulatory functions on the expression of innate immune-related genes (e.g., *CXCL14* and *KIT*). In contrast, no DE miRNAs were identified in Low vs. Control comparison, while both bta-mir-223 and bta-mir-21-3p were reasonably detected to be significantly up-regulated in High vs. Low comparison. The findings here remained consistent with transcriptional and physiological observations that much stronger innate immune responses were induced by high concentration of *S. aureus* relative to low concentration.

### Interactive networks of DEGs in significant BPs and pathways as well as target genes of DE miRNAs

**Figure 4:**
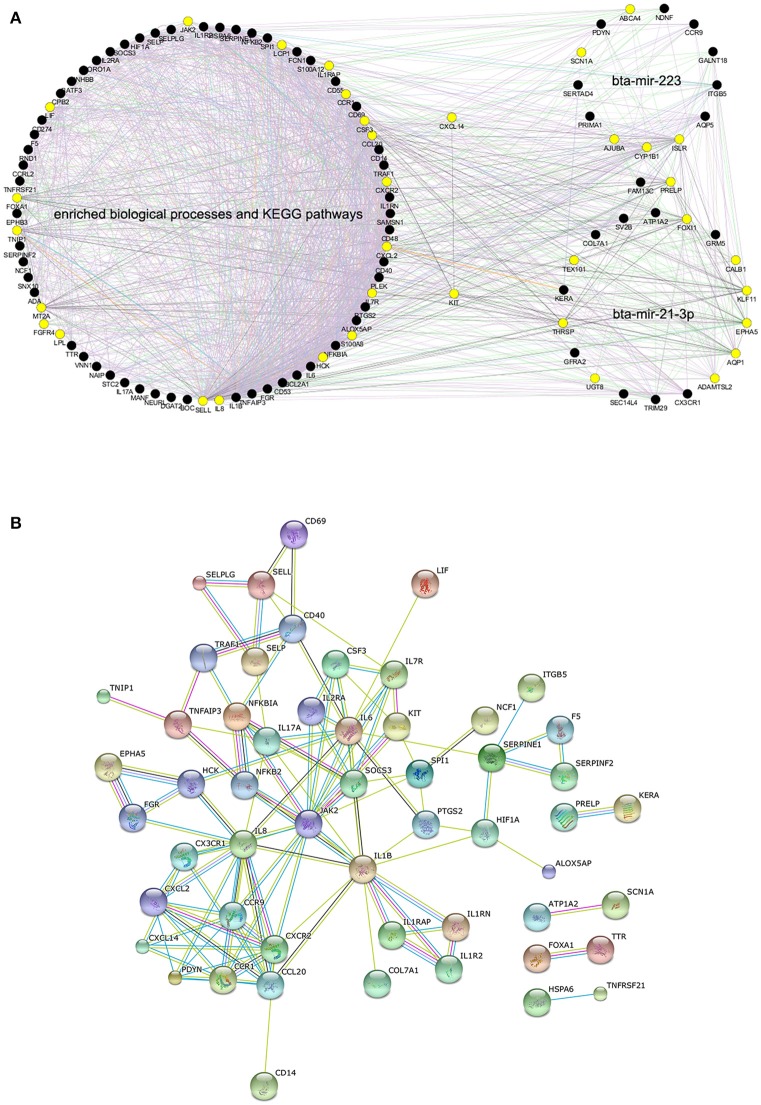
**The predicted interaction networks for genes within enriched biological processes, pathways and candidate targets of differentially expressed miRNAs**. **(A)** Gene-gene interaction network of all the 107 genes produced by GeneMANIA, the yellow solid circles correspond to genes that are directly interconnected with *KIT* or *CXCL14*. **(B)** Protein-protein interaction network of 54 genes produced by STRING.10 (confidence 0.70), as the proteins of the remaining 53 genes were disconnected.

In order to further explore the functional relationship among the significant BP terms, KEGG pathways and DE miRNAs detected in High vs. Control comparison, a total of 107 unique genes was obtained, containing DEGs in the enriched BPs, DEGs in the enriched KEGG pathways and target genes of bta-mir-223 or bta-mir-21-3p (Table S11), as a gene list for the following interaction analyses. The GGI network for all the 107 genes demonstrated that they were highly functionally interconnected (Figure 4A), and the PPI network also shown that a subset of 54 proteins were highly interplayed (Figure 4B). The proteins encoded by *CCR9, CXCL14, CX3CR1, KIT*, and *PDYN*, the target genes of bta-mir-223 or bta-mir-21-3p, were closely interconnected with multiple immune-related proteins in the PPI network, providing supportive evidence that bta-mir-223 and bta-mir-21-3p played crucial roles in regulating the mammary transcriptional response to *S. aureus* infection.

### Validation of expressed genes in bovine mammary gland during *S. aureus* invasion

A total of four genes (*CXCL6, IL8, LPL*, and *RPL10*) were randomly selected from expressed genes for RT-qPCR confirmation. The log-transformed relative expression fold-changes of the four genes in each comparison generated from RT-qPCR were consistent with the results of RNA-Seq analysis (Table 4), and the Pearson correlation between RNA-Seq and RT-qPCR was as high as 0.96, which confirmed the reliability of our analysis.

**Table 4:** **Validation of expressed genes during ***S. aureus*** invasion by real-time quantitative PCR**.

**Ensembl ID**	**Gene name**	**High vs. Control**		**Low vs. Control**		**High vs. Low**	
		**RNA-seq**	**RT-qPCR**	**RNA-seq**	**RT-qPCR**	**RNA-seq**	**RT-qPCR**
**FOLD CHANGE OF GENE EXPRESSION LEVEL ON log**_2_ **SCALE**							
*ENSBTAG00000019716*	*IL-8*	5.85^**^	4.83^**^	2.25	0.91	3.60^**^	3.92^**^
*ENSBTAG00000009812*	*CXCL6*	3.27^**^	2.73^**^	0.77	0.03	2.51^**^	2.36^**^
*ENSBTAG00000012855*	*LPL*	−3.30^**^	−3.77^**^	−1.04	0.30	−1.76	−3.07^*^
*ENSBTAG00000024582*	*RPL10*	0.07	−0.17	0.00	0.03	0.07	−0.61

The numbers with “**” are statistically very significant (for sequencing, FDR < 0.01; for RT-qPCR, P < 0.01); the numbers with “*” are statistically significant (for sequencing, FDR < 0.05; for RT-qPCR, P < 0.05).

### Expression variation of *CXCL14, IL-8, IL-6*, and *JAK2* in Mac-T cells infected by *S. aureus*

**Figure 5:**
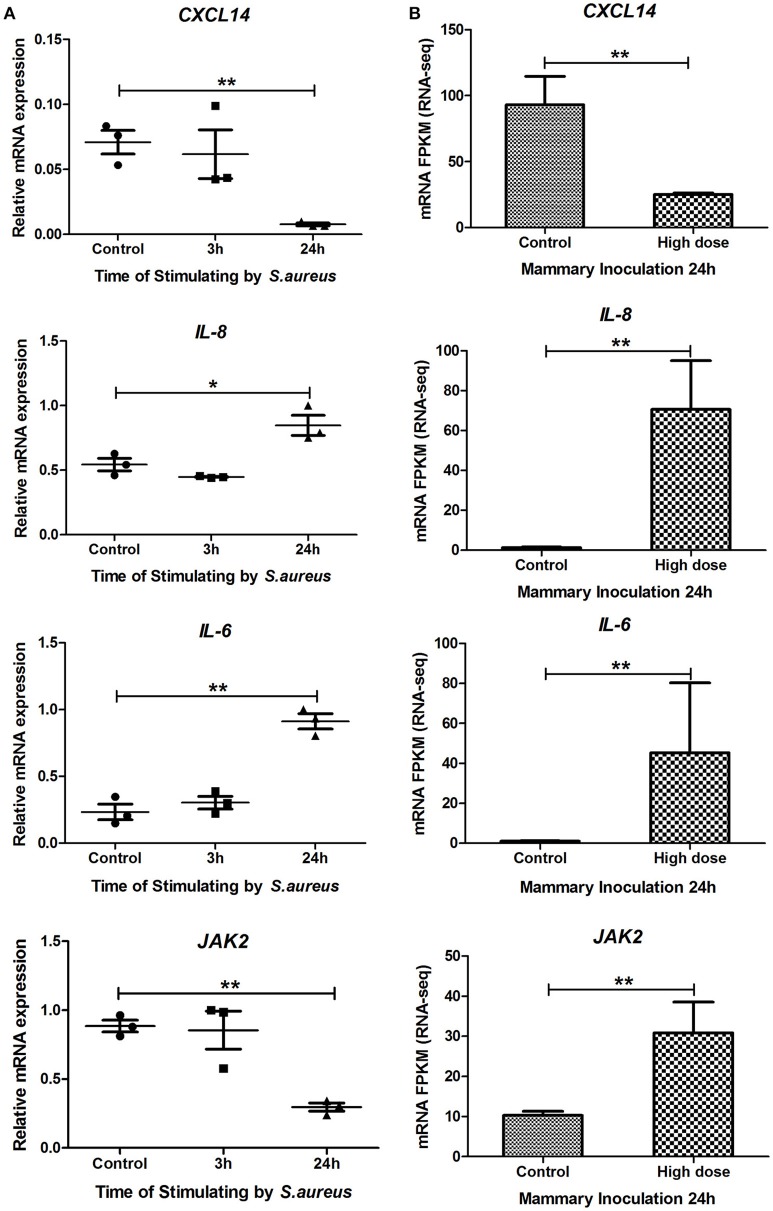
**The mRNA expression levels of ***CXCL14***, ***IL-8***, ***IL-6***, and ***JAK2*** genes in Mac-T cells and RNA-Seq studies. (A)** The mRNA expression levels of *CXCL14, IL-8, IL-6*, and *JAK2* genes in Mac-T cells at 0, 3, and 24 h post-stimulated by *S. aureus*. **(B)** The mRNA expression levels (RPKM-values) of the four genes in mammary tissue at 24 h post IMI with high dose of *S. aureus* and in control group. ^*^ means *P* < 0.05. ^**^ means *P* < 0.01.

Considering the immune functions of *CXCL14, IL-8, IL-6*, and *JAK2* genes and their core positions in PPI network, the relationship between expression levels of these four genes with the progression of *S. aureus* infection was detected using bovine mammary gland epithelial cell line (Mac-T cells) *in vitro*. As shown in Figure 5A, compared to the control, the expression level of *CXCL14* in Mac-T cells was significantly (*P* < 0.01) down-regulated at 24 h after *S. aureus* challenge, and those of *IL-8* and *IL-6* were extremely (*P* < 0.01) up-regulated at 24 h post *S. aureus* infection, which were in line with RNA-Seq analysis *in vivo* (Figure 5B). Although the result of *JAK2* in Mac-T cells at 24 h was contrary to RNA-Seq data, the expression level of *JAK2* was moderately up-regulated at an early treated time point (3 h) in Mac-T cells. The data indicated that the *in vivo* and *in vitro* regulation of *JAK2* gene expression differs in kinetics after *S. aureus* infection.

### Integrated analysis of DEGs, target genes of DE miRNAs, and previously reported disease susceptibility and mastitis-related QTLs

All the DEGs detected in the present study were interspersed throughout the bovine genome (Figure S3), providing supportive evidence that bovine mastitis follows a polygenic model of inheritance. In order to gain a systematic insight into the association of the DEGs and target genes of DE miRNAs with disease susceptibility/mastitis-related traits and better understand the genetic and biological basis underlying such complex traits, a total of 225 unique genes, containing DEGs and target genes of DE miRNAs detected in High vs. Control group together with 1659 previously reported disease susceptibility/mastitis-related QTLs (http://www.animalgenome.org/cgi-bin/QTLdb/BT/index), were integrated by comparing their genomic locations based on the reference genome UMD3.1. Genes with start and end positions within or across the QTL regions were considered as successfully annotated in QTLs.

In this context, 1528 out of all the 24,616 Ensembl genes were successfully annotated into the 1659 QTLs, and 28 out of the studied 225 genes in this integrated analysis were located in 22 QTLs (Table S12). Based on a classical hypergeometric enrichment test implemented in R, DEGs and target genes of DE miRNAs were significantly enriched in disease susceptibility/mastitis-related QTLs (*P* = 1.44 × 10^−4^), inferring that the transcriptionally active regions induced by IMI tended to be strongly associated with bovine mastitis. More interestingly, out of these 28 genes successfully annotated in the disease susceptibility/mastitis-related QTLs, *AJUBA, AQP5, CXCL14*, and *SCN1A* were target genes of bta-mir-223, while *KERA, TEX101*, and *TRIM29* were target genes of bta-mir-21-3p. The detailed information for the integrated analysis was summarized in Table 5. Compared with genes that are located in QTLs do not show differential expression, genes with differential expression are more likely to be engaged in traits-related biological processes and thus more likely to be truly associated with the complex traits. Therefore, these 28 genes annotated in the disease susceptibility/mastitis-related QTLs were suggested as candidate genes for *S. aureus* mastitis in dairy cattle.

**Table 5:** **Detailed information on the previous QTLs containing DEGs and target genes of DE miRNAs within High vs. Control comparison**.

**Gene name**	**Gene position**	**QTL_ID**	**QTL position**	**Trait**
*ENSBTAG00000010852*	23:31342479_31374714	30570	23:30452407_32030774	AMI
*AJUBA*	10:21699229_21708079	19933	10:21023960_21977581	BSE
*SCN1A*	2:30321635_30415283	37297	2:30080851_32170510	BVDV
*TRIM29*	15:31213143_31240221	37307	15:30080851_32170510	BVDV
*CCDC65*	5:31052620_31060822	30587	5:30452407_32030774	CMI
*RND1*	5:31090624_31097555	30587	5:30452407_32030774	CMI
*ENSBTAG00000039992*	5:30728351_30735852	30587	5:30452407_32030774	CMI
*ENSBTAG00000037775*	5:30713143_30723571	30587	5:30452407_32030774	CMI
*NRCAM*	4:49523461_49840744	19008	4:46178647_52998234	CM
*SLC26A4*	4:48955564_49016104	19008	4:46178647_52998234	CM
*CXCL14*	7:48661077_48669442	19014	7:46178647_52998234	CM
*SLC4A11*	13:52436709_52447922	19017	13:46178647_52998234	CM
*SCNN1D*	16:52594295_52599757	19023	16:46178647_52998234	CM
*ENSBTAG00000035926*	12:707017_708407	24036	12:93479_906131	IBK
*PLAUR*	18:52202331_52215619	18838	18:46178647_52998234	MP
*TEX101*	18:51897363_51900497	18838	18:46178647_52998234	MP
*ENSBTAG00000006859*	18:51735955_51818445	18838	18:46178647_52998234	MP
*IL6*	4:31578310_31582667	37310	4:30080851_32170510	MP
*GSAP*	4:44061013_44150547	31626	4:44067070_44085642	SCC
*RRAD*	18:34746054_34749250	18469	18:11438802_46178647	SCS
*ENSBTAG00000023659*	18:24125362_24128164	18469	18:11438802_46178647	SCS
*ENSBTAG00000038706*	18:24112350_24114381	18469	18:11438802_46178647	SCS
*PLAUR*	18:52202331_52215619	18470	18:46178647_52983181	SCS
*TEX101*	18:51897363_51900497	18470	18:46178647_52983181	SCS
*ENSBTAG00000006859*	18:51735955_51818445	18470	18:46178647_52983181	SCS
*IRG1*	12:52415727_52425420	19034	12:46178647_52998234	SCS
*FASN*	19:51384523_51403614	19038	19:46178647_52998234	SCS
*FOXA1*	21:48212805_48224542	19042	21:46178647_52998234	SCS
*AQP5*	5:30080019_30085113	20620	5:18046673_47449338	SCS
*NFE2*	5:25928792_25936085	20620	5:18046673_47449338	SCS
*CCDC65*	5:31052620_31060822	20620	5:18046673_47449338	SCS
*RND1*	5:31090624_31097555	20620	5:18046673_47449338	SCS
*KERA*	5:20995380_21002991	20620	5:18046673_47449338	SCS
*ENSBTAG00000039992*	5:30728351_30735852	20620	5:18046673_47449338	SCS
*ENSBTAG00000037775*	5:30713143_30723571	20620	5:18046673_47449338	SCS
*KERA*	5:20995380_21002991	32241	5:20001875_20999270	SCS
*GSAP*	4:44061013_44150547	40359	4:44050471_44087629	SCS

*AMI, Antibody-mediated immune response; BSE, Bovine spongiform encephalopathy; BVDV, Bovine viral diarrhea virus susceptibility; CMI, Cell-mediated immune response; CM, Clinical mastitis; IBK, Infectious bovine keratoconjunctivitis susceptibility; MP, M. paratuberculosis susceptibility; SCC, Somatic cell count; SCS, Somatic cell score*.

## Discussion

Mastitis induced by *S. aureus* is resistant to antibiotic treatment and very difficult to be prevented and eliminated. This study simultaneously investigated the transcriptional and post-transcriptional regulatory responses of the bovine mammary gland to IMI with *S. aureus* using the next-generation sequencing technology. Our results demonstrated that several important innate immune and inflammatory-relevant pathways and BPs were significantly impacted during IMI with high concentration of *S. aureus*. Notably, both transcriptional and post-transcriptional responses of bovine mammary gland to invading *S. aureus* were dosage-dependent and regulated by a complicated interplay of gene-miRNA-pathway.

### Transcriptional responses of mammary gland to *S. aureus*

A previous study profiled the expression of 1480 immune-related genes in bovine mammary gland at 16 h post IMI with *S. aureus* (strain JG80, 5 × 10^2^–10^6^ cfu/mL) using the Bovine Innate Immune Microarray, where they found that 55 genes were up-regulated (FDR < 0.05) (Lutzow et al., 2008). Regardless of the differences between the previous study and the current study (e.g., different animals, infection doses, challenge times, and platforms), 13 out of these 55 up-regulated genes were also detected to be significantly up-regulated in this study during IMI with high concentration of *S. aureus*, including genes for cytokine, chemokine and intercellular signaling (*CCL20, CSF3, HCK, IL1B, IL6, PLAUR*, and *S100A12*), cell surface receptors (*CD14* and *CD40*), transcription regulator (*NFKB2* and *NFKBIA*), and apoptosis (*BCL2A1* and *BIRC3*). Compared to the previous study, except that much more innate immune-related genes were observed as up-regulated, several genes engaged into monocarboxylic acid biosynthetic processes and lipid metabolism were observed as highly down-regulated in udder quarters infected with high concentration of *S. aureus*. It is well-known that cows with mastitis experience reduced milk production (Gröhn et al., 2004), however, further research is needed to explore whether these down-regulated genes actually affect the protein or fat synthesis in mammary gland.

More recently, another study exploited the transcriptional responses of Mac-T cells to the infection with *S. aureus in vitro* using RNA-Seq (Wang et al., 2013). Two significantly enriched KEGG pathways were shared by their study and the present study (FDR < 0.05), NOD-like receptor signaling pathway and chemokine signaling pathway. NOD-like receptor family is responsible for detecting pathogens and generating innate immune responses, and it acts in concert with Toll-like receptor signaling pathway that also plays a crucial role in recognizing pathogen-associated molecular patterns and inducing innate immunity (Fritz et al., 2006; Kanneganti et al., 2007). Toll-like receptor signaling pathway is mainly represented by *CD14* (Barton and Medzhitov, 2003) that was detected as significantly up-regulated [log_2_(fold-change) = 2.2] in the present study (Table S7). The up-regulation of *CD14* might induce the up-regulation of down-stream genes (e.g., *NFKBIA, IL1B, IL8, CD40*, and *IL6*) in Toll-like receptor signaling pathway as observed in the current study (FDR = 0.06; Figure S4), however, it remains to be shown whether and how these down-stream genes are activated by the up-regulation of *CD14* in the further research. Chemokine signaling pathway plays important roles in regulating and mobilizing immune-relevant cells, and is involved in providing directional cues for leukocytes to the site of inflammation (Mellado et al., 2001), indicating the importance of recruitment and activation of immune cells to sites of infection during acute *S. aureus* mastitis. Noticeably, several other pathways primarily involved in pro-inflammatory and innate immune system were uncovered in the present study but not in Wang et al. (2013), including cytokine-cytokine receptor interaction, Jak-STAT signaling pathway (FDR = 0.05; Figure S5) and Toll-like receptor signaling pathway, all of which were also demonstrated to be associated with *S. aureus* mastitis in both dairy cows and goats by previous studies (Pisoni et al., 2010; He et al., 2016). However, several human neurological disease-related pathways (e.g., Huntingson's, Alzheimer's, and Parkinson's diseases) were only observed in Wang et al. (2013). The findings here and in Wang et al. (2013) indicate that the transcriptional responses of the mammary gland to IMI with *S. aureus in vivo* could be different from responses of Mac-T cells *in vitro* due to the complex *in vivo* environment and the interplays among various cell types.

Two infectious diseases-related pathways (i.e., legionellosis and malaria pathways) were the first time to be reported to be associated with *S. aureus* invasion in the present study. These two pathways have been reported to be relevant with *Escherichia coli* (*E. coli*) and *Streptococcus uberis* (*S. uberis*) infections in dairy cattle (Loor et al., 2011; Bionaz et al., 2012), inferring that bovine innate immue responses to various invading pathogens could share certain evolutionaly conservation.

### Post-transcriptional responses of mammary gland to *S. aureus*

The expression of 14 immune-related miRNAs has been investigated previously in bovine mammary gland responsive to IMI with *S. uberis* using RT-qPCR (Naeem et al., 2012). The up-regulation of bta-mir-223 was observed, which was consistent with the present study. The biological roles of mir-223 include inhibition of cell cycle progression, negative regulation of neutrophil proliferation, and granulocyte differentiation (Laios et al., 2008), and it has also been identified as sensitive and specific biomarkers for sepsis defined as the combination of infection and inflammatory response syndrome (Wang et al., 2010). Additionally, it has been reported that the up-regulation of mir-223 was partly controlled by the up-regulation of *CEBPE* (CCAAT enhancer binding protein) both in human and bovine studies (Fazi et al., 2005; Moyes et al., 2009). Human and mice lacking active CEBPE protein always suffer from frequent bacterial infections due to functionally defective neutrophils and macrophages (Gombart et al., 2005). In the present study *CEBPE* was also detected as significantly up-regulated [log_2_(fold-change) = 3] (Table S7). These findings indicate that *CEBPE* could play a regulatory role in bovine mammary responses to IMI through regulating the expression of bta-mir-223, which in turn post-transcriptionally regulates the expression of other important immune-related genes. However, it remains to be investigated whether and how the expression of bta-mir-223 is regulated by *CEBPE* gene.

Recently, bta-mir-21-3p has been reported to be significantly up-regulated (*P* < 0.05) in bovine Mac-T cells at 24 h post *S. aureus* infection (Jin et al., 2014), which is in line with the present study. The up-regulation of bta-mir-21-3p has also been observed at 24 h post-infection with *Mycobacterium bovis* in the alveolar macrophage (Vegh et al., 2015). It has been proposed that mir-21-3p negatively regulates the vitamin D-dependent antimicrobial pathway during infection with *Mycobacterium leprae* (O'Neill et al., 2011; Liu et al., 2012). Vitamin D-dependent antimicrobial pathway is biologically critical for the response of the innate immune system to infection and wounds, and the deficiency of it leads to suboptimal response to bacterial infections (Gombart, 2009). *CALB1*, a target gene of bta-mir-21-3p in the present study, is engaged in vitamin D binding biological process (Wojtusik and Johnson, 2012), which might indicate that mir-21-3p impacted vitamin D-dependent antimicrobial pathway partly through post-transcriptionally down-regulating *CALB1*. Further research is needed to explore the functional relationship among *CALB1*, bta-mir-21-3p and vitamin D binding biological process.

### Network analysis of DEGs and target genes of DE miRNAs

**Figure 6:**
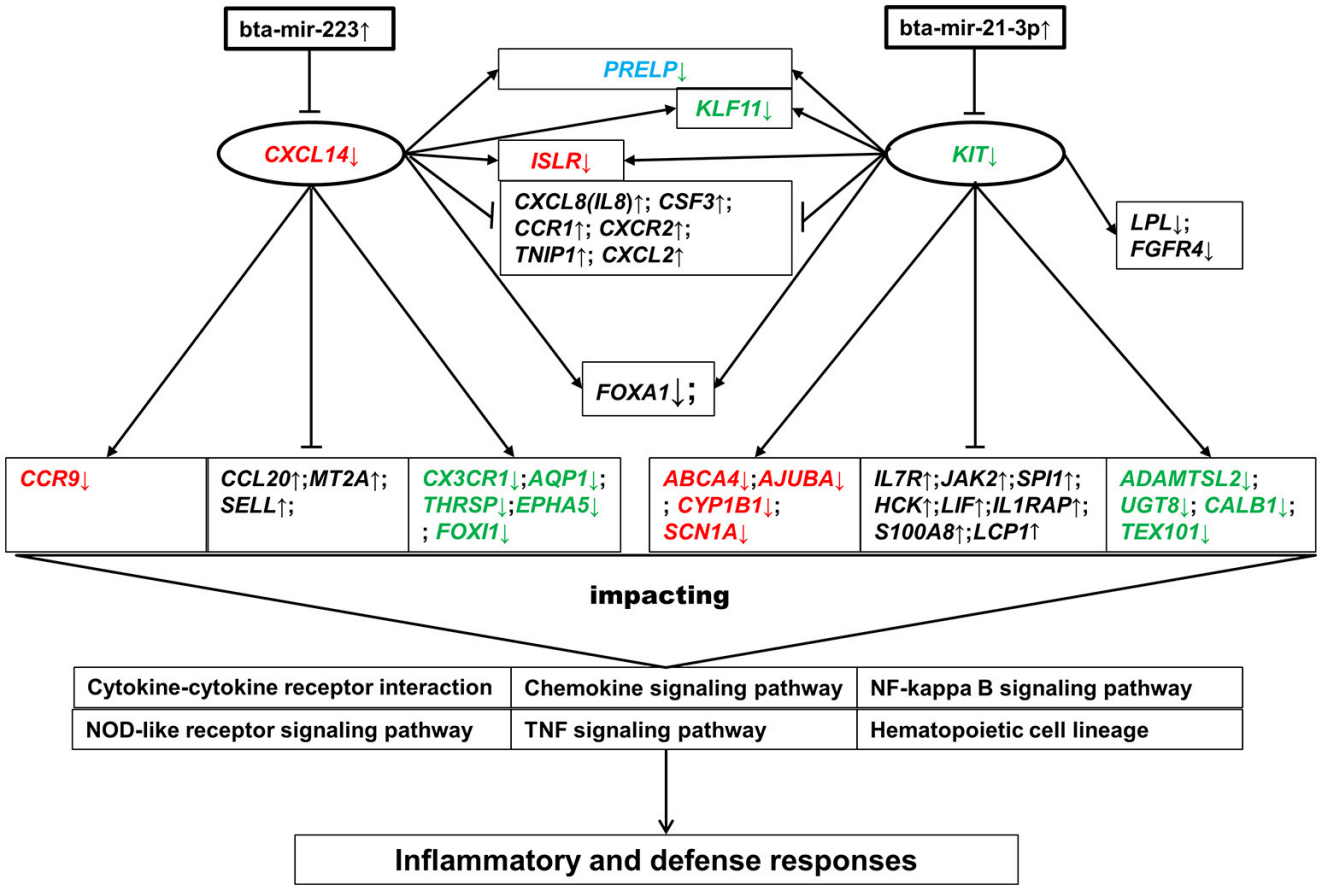
**Gene-pathway interactive network modulated by bta-mir-223 and bta-mir-21-3p**. Genes in red are the target genes of bta-mir-223. Genes in green are the target genes of bta-mir-21-3p. The gene in blue is a common target gene of both bta-mir-223 and bta-mir-21-3p. Genes in black are not the target genes of either bta-mir-223 or bta-mir-21-3p. ↑ represents up-regulation, and ↓ represents down-regulation. The lines between genes represent direct interconnection based on the PPI and GGI networks: arrow- and end-lines represent positive and reverse regulation in this study, respectively.

Of most interest were the two target genes of bta-mir-223 and bta-mir-21-3p, *CXCL14* and *KIT*, which were shared by the enriched BPs and KEGG pathways and were also structurally presented as the pivotal nodes in both GGI and PPI networks (Figure 4). *CXCL14* has been proposed to be involved in the homeostasis of monocyte-derived macrophages, and to be down-regulated upon transition to malignancy (Kurth et al., 2001; Starnes et al., 2006). *KIT* controls many intracellular signal transduction pathways that regulate cell proliferation and apoptosis, and its deregulation impairs various cellular physiological functions, leading to serious human diseases (Chauvot de Beauchêne et al., 2014; Stankov et al., 2014). Based on the direct interaction in PPI and GGI networks, the down-regulation of *CXCL14* and *KIT* might directly influence the expression of multiple innate immune-related genes (e.g., *CX3CR1, CXCR2, CCR9, CCR1, SPI1, JAK2, IL1RAP, IL7R*, and *CSF3*) and lipid-metabolism-related genes (e.g., *LPL*) as well as other target genes of bta-mir-223 and bta-mir-21-3p (e.g., *AQP1, ADAMTSL2, SCN1A*, and *ISLR*) (Figure 6). These impacted genes then affected various inflammatory-related biological processes and pathways (e.g., cytokine-cytokine receptor interaction and chemokine signaling pathway) through implicating the expression of the relevant down-stream genes, resulting in host inflammatory and defense responses (Figure 6). These findings could explain how the deregulation of some key “hub” miRNAs or genes might influence multiple important pathways leading to phenotypic changes, and also confirmed that the response of the mammary gland to *S. aureus* infection was regulated by a complex network of miRNA-gene-pathway interplay.

## Conclusion

The present study provided a global view of transcriptional and post-transcriptional regulatory responses in the bovine mammary gland to IMI with different concentrations of *S. aureus in vivo*, and demonstrated that the responses of the mammary gland to *S. aureus* were dosage-dependent. Several innate immune and inflammatory-relevant pathways and BPs were significantly impacted during IMI with high concentration of *S. aureus*, such as cytokine-cytokine receptor interaction and inflammatory response. Bta-mir-223 and bta-mir-21-3p were likely to be the central post-transcriptional regulators of the innate immune response to IMI with *S. aureus* in bovine mammary gland through regulating the expression of some key immune-related genes (e.g., *CXCL14* and *KIT*), which demonstrated that the response to *S. aureus* in bovine mammary gland was regulated by a miRNA-gene-pathway network. The significant down-regulation of *CXCL14* was also observed in bovine mammary epithelial cells at 24 h post-infection with high concentration of *S. aureus in vitro*. A total of 28 genes (e.g., *CXCL14, KIT*, and *SLC4A11*) were suggested as the most promising candidates associated with *S. aureus*-induced mastitis for follow-up functional studies.

## Author contributions

YY conceived the experiment, XW, CL, YD, HZ, JA, and YY collected samples, LF and YD performed RT-qPCR, LF, PS, YW, BL, SZ, and YY designed the study, LF and YH analyzed the results and wrote the manuscript. All authors reviewed and contributed to the manuscript.

## Funding

This research was financially supported by the National Natural Science Foundation of China (31272420 and 31301963), Basic Research from the Ministry of Education of the People's Republic of China (2011JS006), Modern Agro-industry Technology Research System (CARS-37), the Twelfth Five-Year plan of National Science and Technology Project in Rural Areas (2011BAD28B02), State High-Tech Development Plan of China (2008AA101002), the Program for Changjiang Scholar and Innovation Research Team in University (IRT1191), and Youth Innovation Promotion Association, Chinese Academy of Sciences.

### Conflict of interest statement

The authors declare that the research was conducted in the absence of any commercial or financial relationships that could be construed as a potential conflict of interest.

## Supplementary material

Figure S1 **The summary of the bioinformatics analyses strategy**.

Figure S2 **The expression correlations for all the profiled 358 known mature miRNAs between each pair of samples**.

Figure S3 **The distribution of differentially expressed genes (DEG) along the bovine genome**. y-Axis is the chromosome number, x-axis is the base-pair position of DEG.

Figure S4 **Differentially expressed genes (DEG) in Toll-like receptor signaling pathway**. The colored genes are DEGs, the color-key corresponds to the log_2_(fold-change) of DEG.

Figure S5 **Differentially expressed genes (DEG) in JAK-STAT signaling pathway**. The colored genes are DEGs, the color-key corresponds to the log_2_(fold-change) of DEG.
