# Supplementary figures and images for: Genome-Wide Transcriptional and Post-transcriptional Regulation of Innate Immune and Defense Responses of Bovine Mammary Gland to Staphylococcus aureus
Source: Front Cell Infect Microbiol. 2016 Dec 26;6:193. doi: 10.3389/fcimb.2016.00193 (PMC5183581; doi:10.3389/fcimb.2016.00193)

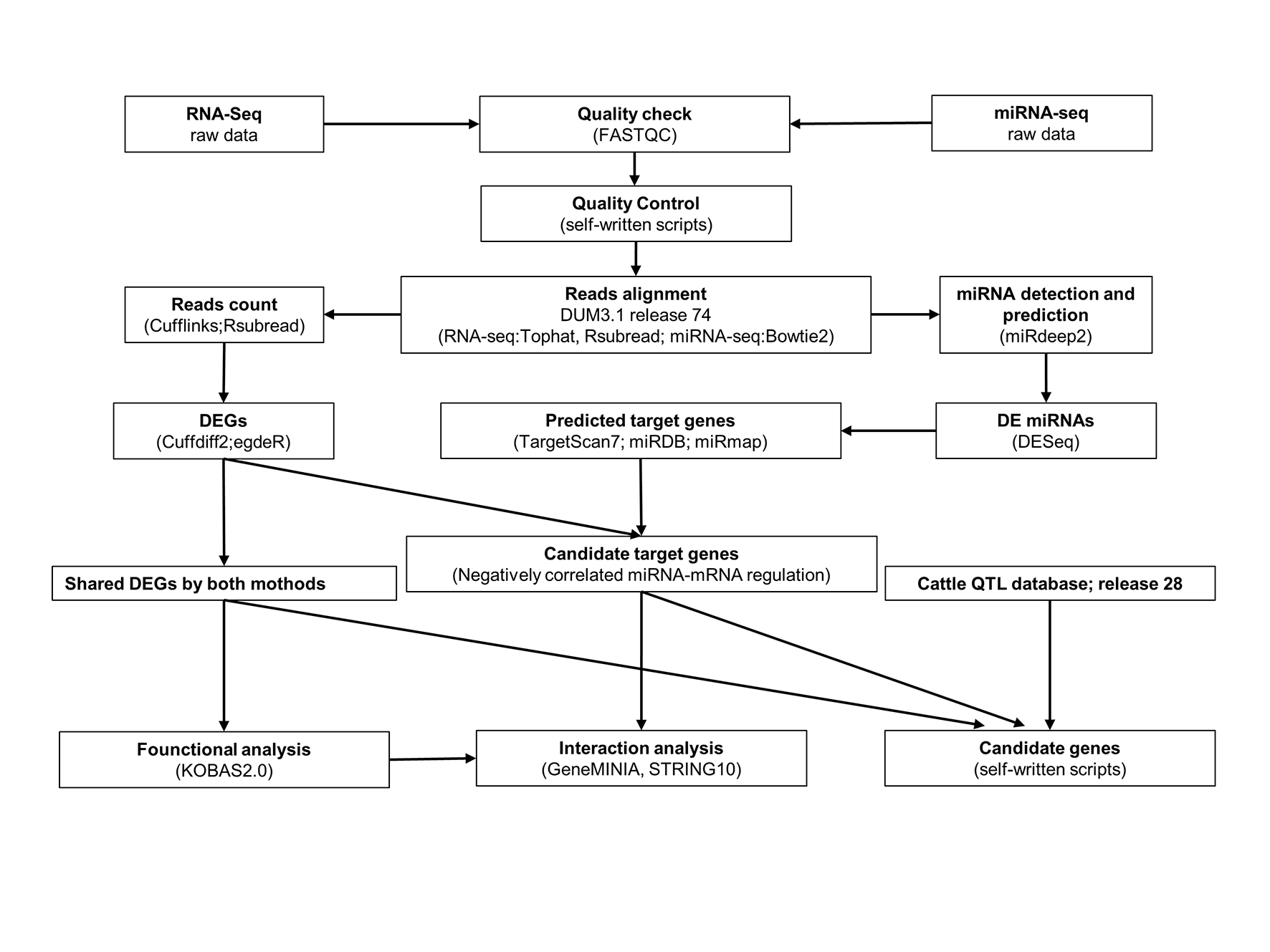

Supplement: Figure S1 — The summary of the bioinformatics analyses strategy. [file Image1.tif]

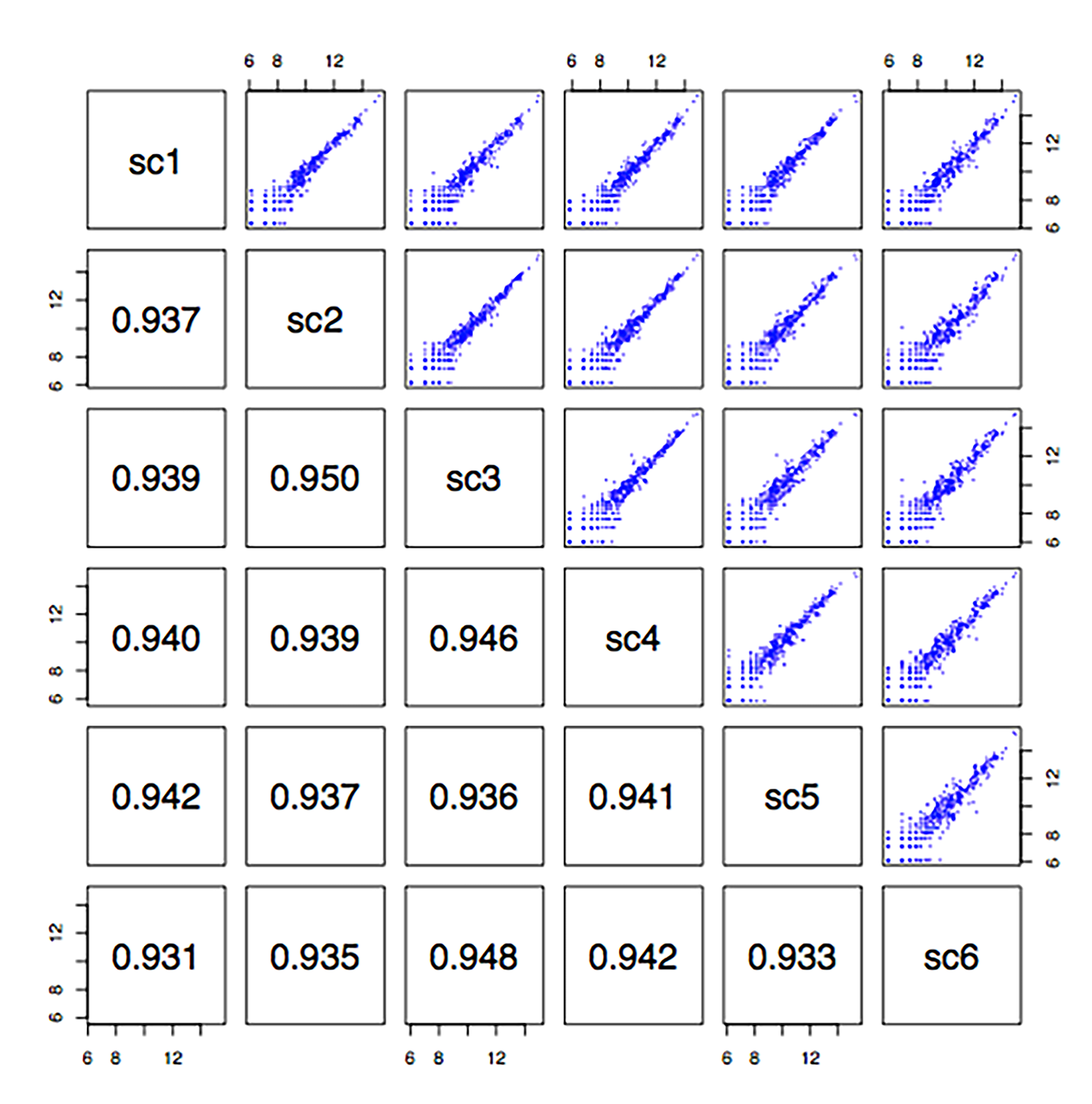

Supplement: Figure S2 — The expression correlations for all the profiled 358 known mature miRNAs between each pair of samples. [file Image2.tif]

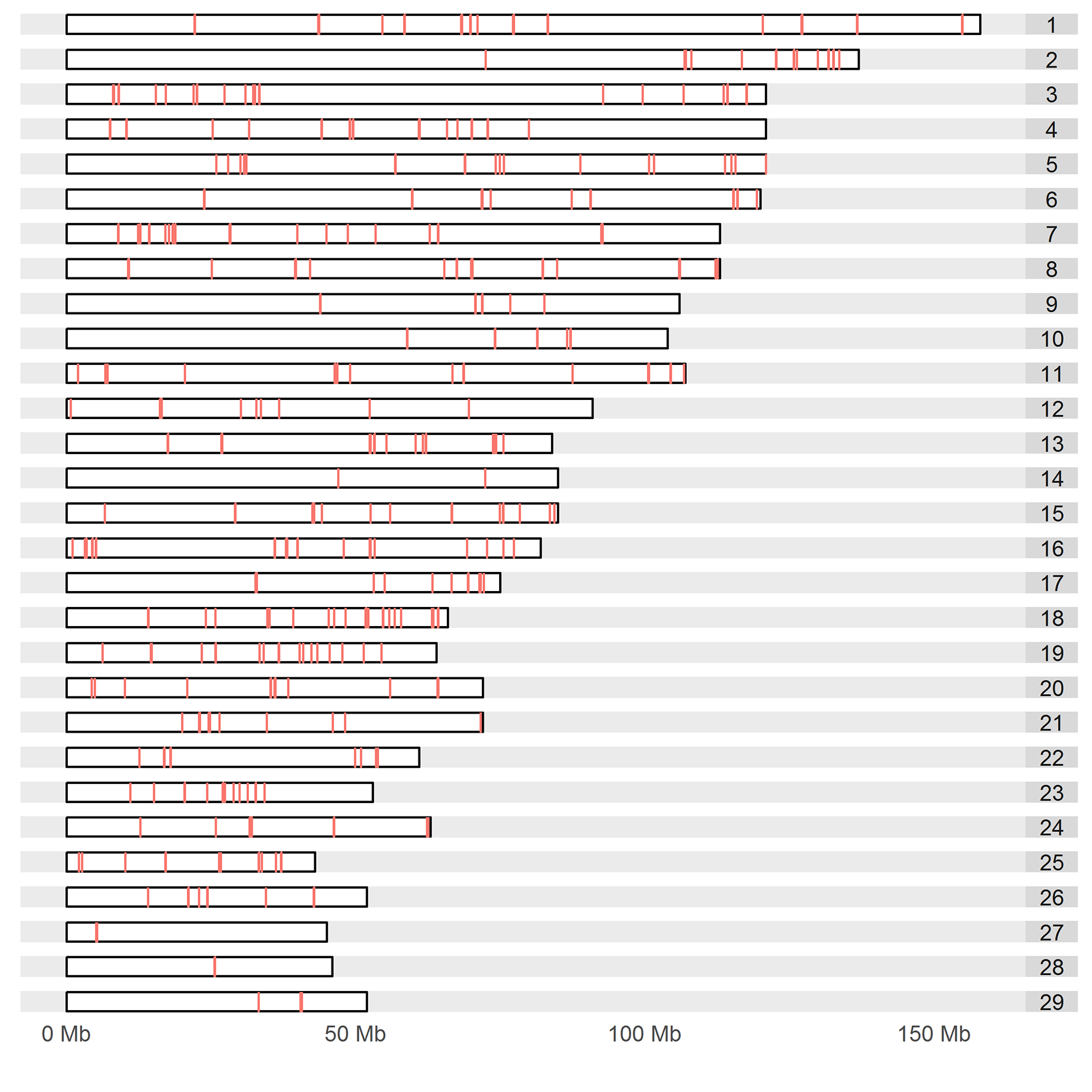

Supplement: Figure S3 — The distribution of differentially expressed genes (DEG) along the bovine genome. y-Axis is the chromosome number, x-axis is the base-pair position of DEG. [file Image3.tiff]

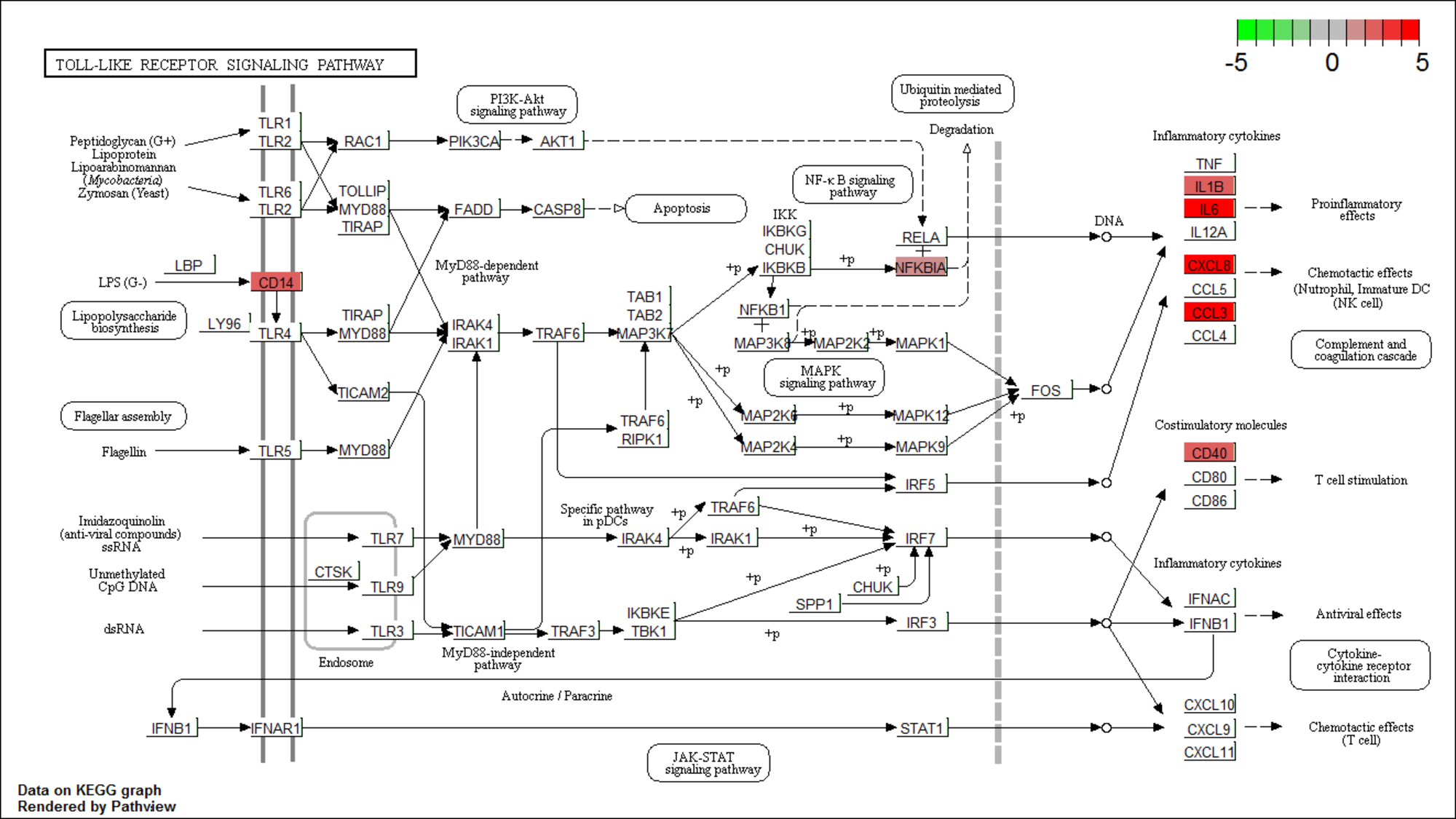

Supplement: Figure S4 — Differentially expressed genes (DEG) in Toll-like receptor signaling pathway. The colored genes are DEGs, the color-key corresponds to the log2(fold-change) of DEG. [file Image4.jpg]

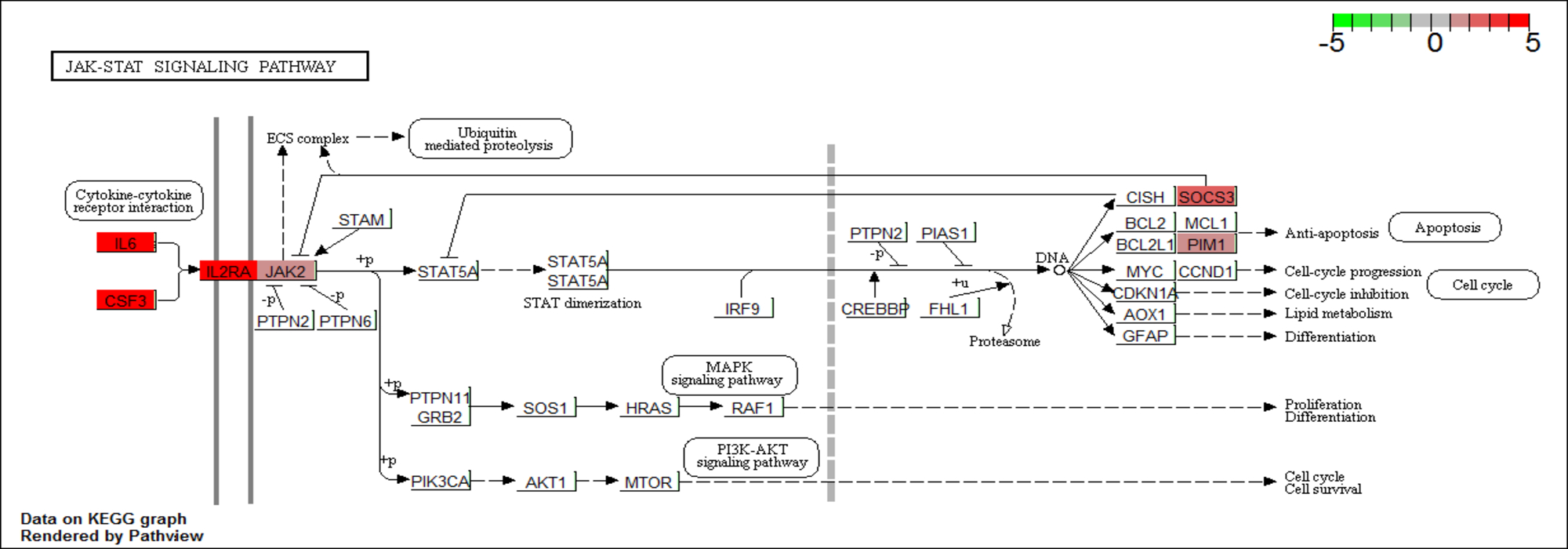

Supplement: Figure S5 — Differentially expressed genes (DEG) in JAK-STAT signaling pathway. The colored genes are DEGs, the color-key corresponds to the log2(fold-change) of DEG. [file Image5.jpg]
