# Supplementary material for: Genome-Wide Transcriptional and Post-transcriptional Regulation of Innate Immune and Defense Responses of Bovine Mammary Gland to Staphylococcus aureus
Source: Front Cell Infect Microbiol. 2016 Dec 26;6:193. doi: 10.3389/fcimb.2016.00193 (PMC5183581; doi:10.3389/fcimb.2016.00193)
Supplement: Supplementary file 7 [file Table2.docx]

**Table S2. The basic statistics for RNA-seq reads generated from mammary glands of each sample and the subsequent alignment information**

| Software | Mapping summary | Cow_A/sample1  (control1) | Cow_B/sample2  (control2) | Cow_A/sample3  (low1) | Cow_B/sample4  (low2) | Cow_A/sample5  (high1) | Cow_B/sample6  (high2) |
| --- | --- | --- | --- | --- | --- | --- | --- |
| Tophat | Clean reads | 52,614,666 | 53,514,884 | 51,049,424 | 50,013,352 | 51,405,852 | 64,092,924 |
|  | Total mapped reads | 49,455,822 | 46,537,318 | 45,757,580 | 44,534,074 | 45,786,232 | 25,040,876 |
|  | Uniquely mapped reads | 45,596,902 | 46,192,016 | 43,025,316 | 41,943,718 | 42,829,232 | 23,361,594 |
|  | Multiple mapped reads | 3,858,920 | 345,302 | 2,732,264 | 2,590,356 | 2,957,000 | 1,679,282 |
|  | Uniquely Mapping rate | 86.67% | 86.32% | 84.28% | 83.87% | 83.32% | 36.45% |
| Rsubread | Uniquely mapped reads | 48,715,919 | 49,820,822 | 46,579,843 | 45,440,671 | 46,938,190 | 25,255,124 |
|  | Uniquely Mapping rate | 92.59% | 93.10% | 91.24% | 90.86% | 91.31% | 39.40% |
