# Supplementary material for: Genome-Wide Transcriptional and Post-transcriptional Regulation of Innate Immune and Defense Responses of Bovine Mammary Gland to Staphylococcus aureus
Source: Front Cell Infect Microbiol. 2016 Dec 26;6:193. doi: 10.3389/fcimb.2016.00193 (PMC5183581; doi:10.3389/fcimb.2016.00193)
Supplement: Supplementary file 8 [file Table3.docx]

**Table S3. The basic statistics for miRNA-seq reads generated from mammary glands and the subsequent alignment information**

| Mapping summary | Cow_A/sample1  (control1) | Cow_B/sample2  (control2) | Cow_A/sample3  (low1) | Cow_B/sample4  (low2) | Cow_A/sample5  (high1) | Cow_B/sample6  (high2) |
| --- | --- | --- | --- | --- | --- | --- |
| Clean reads | 226,001 | 443,902 | 323,655 | 184,724 | 332,660 | 366,724 |
| mapped reads | 125,966 | 292,034 | 196,191 | 104,856 | 216,335 | 230,155 |
| unmapped reads | 100,035 | 151,868 | 127,464 | 79,868 | 116,325 | 136,569 |
| Mapping rate | 55.7% | 65.8% | 60.6% | 56.8% | 65.0% | 62.8% |
