# Supplementary material for: Genome-Wide Transcriptional and Post-transcriptional Regulation of Innate Immune and Defense Responses of Bovine Mammary Gland to Staphylococcus aureus
Source: Front Cell Infect Microbiol. 2016 Dec 26;6:193. doi: 10.3389/fcimb.2016.00193 (PMC5183581; doi:10.3389/fcimb.2016.00193)
Supplement: Supplementary file 15 [file Table10.docx]

**Table S10.** The target genes of bta-mir-223 and bta-mir-21-3p in High *vs*. Control comparison

| bta-mir-223 | Ensembl ID | Gene name | Log2FC_Cuffdiff2 | *FDR*_Cuffdiff2 | Log2FC_edgeR | *FDR*_edgeR |
| --- | --- | --- | --- | --- | --- | --- |
|  | *ENSBTAG00000006694* | *CXCL14* | -1.89 | 5.77E-03 | -1.94 | 3.27E-03 |
|  | *ENSBTAG00000026813* | *AQP5* | -3.49 | 5.77E-03 | -3.07 | 1.31E-02 |
|  | *ENSBTAG00000002914* | *GALNT18* | NA | NA | -1.85 | 4.90E-02 |
|  | *ENSBTAG00000006024* | *ISLR* | NA | NA | -4 | 5.86E-03 |
|  | *ENSBTAG00000010531* | *CYP1B1* | NA | NA | -2.03 | 4.30E-02 |
|  | *ENSBTAG00000010634* | *NDNF* | NA | NA | -2.84 | 1.54E-04 |
|  | *ENSBTAG00000010850* | *SERTAD4* | NA | NA | -1.78 | 6.23E-03 |
|  | *ENSBTAG00000012724* | *AJUBA* | NA | NA | -1.42 | 4.10E-02 |
|  | *ENSBTAG00000013755* | *ITGB5* | -1.51 | 4.04E-02 | NA | NA |
|  | *ENSBTAG00000015880* | *PRIMA1* | NA | NA | -2.41 | 2.02E-03 |
|  | *ENSBTAG00000017540* | *FAM13C* | NA | NA | -2.45 | 3.13E-04 |
|  | *ENSBTAG00000017834* | *PRELP* | -2.12 | 4.64E-02 | NA | NA |
|  | *ENSBTAG00000018010* | *ABCA4* | NA | NA | -5.23 | 1.89E-14 |
|  | *ENSBTAG00000018520* | *SCN1A* | NA | NA | -3.58 | 3.33E-02 |
|  | *ENSBTAG00000031348* | *CCR9* | NA | NA | -2.04 | 1.54E-02 |
|  | *ENSBTAG00000045520* | *PDYN* | NA | NA | -6.08 | 2.18E-02 |
|  | *ENSBTAG00000002699* | *KIT* | -1.46 | 4.28E-02 | -1.68 | 3.65E-02 |
|  | *ENSBTAG00000031351* | *SEC14L4* | -2.71 | 3.15E-02 | -2.21 | 3.85E-03 |
| bta-mir-21-3p | *ENSBTAG00000000745* | *AQP1* | -2.03 | 5.77E-03 | NA | NA |
|  | *ENSBTAG00000002923* | *CX3CR1* | -2.24 | 3.61E-02 | NA | NA |
|  | *ENSBTAG00000004574* | *UGT8* | NA | NA | -3.36 | 8.27E-06 |
|  | *ENSBTAG00000007492* | *ADAMTSL2* | NA | NA | -3 | 2.84E-04 |
|  | *ENSBTAG00000009419* | *TRIM29* | -2.01 | 1.01E-02 | NA | NA |
|  | *ENSBTAG00000009438* | *EPHA5* | NA | NA | -4.32 | 8.14E-05 |
|  | *ENSBTAG00000010551* | *ATP1A2* | NA | NA | -2.92 | 1.44E-02 |
|  | *ENSBTAG00000011666* | *THRSP* | NA | NA | -2.83 | 9.65E-03 |
|  | *ENSBTAG00000014340* | *KERA* | NA | NA | -2.38 | 4.68E-02 |
|  | *ENSBTAG00000014620* | *FOXI1* | NA | NA | -4.26 | 1.87E-06 |
|  | *ENSBTAG00000015924* | *CALB1* | NA | NA | -7.9 | 3.37E-02 |
|  | *ENSBTAG00000016777* | *TEX101* | NA | NA | -4.28 | 1.21E-02 |
|  | *ENSBTAG00000017540* | *FAM13C* | NA | NA | -2.45 | 3.13E-04 |
|  | *ENSBTAG00000017834* | *PRELP* | -2.12 | 4.64E-02 | NA | NA |
|  | *ENSBTAG00000019081* | *COL7A1* | -2.12 | 1.40E-02 | NA | NA |
|  | *ENSBTAG00000020665* | *GFRA2* | NA | NA | -1.95 | 1.14E-02 |
|  | *ENSBTAG00000027074* | *SV2B* | NA | NA | -3.13 | 1.29E-02 |
|  | *ENSBTAG00000046218* | *KLF11* | NA | NA | -2.09 | 4.20E-04 |
|  | *ENSBTAG00000048061* | *GRM5* | NA | NA | -3.01 | 2.78E-03 |

NA: no values; FC: fold-change; FDR: false discovery rate.
